# Complete genome of Rhizobium *leguminosarum* Norway, an ineffective *Lotus* micro-symbiont

**DOI:** 10.1186/s40793-018-0336-9

**Published:** 2018-12-05

**Authors:** Juan Liang, Anne Hoffrichter, Andreas Brachmann, Macarena Marín

**Affiliations:** Institute of Genetics, Faculty of Biology, Ludwig-Maximilians-University Munich, Munich, Germany

**Keywords:** Symbiosis, *Rhizobium*, Legume, Ineffective nodulation, Genome

## Abstract

Rhizobia bacteria engage in nitrogen-fixing root nodule symbiosis, a mutualistic interaction with legume plants in which a bidirectional nutrient exchange takes place. Occasionally, this interaction is suboptimal resulting in the formation of ineffective nodules in which little or no atmospheric nitrogen fixation occurs. *Rhizobium leguminosarum* Norway induces ineffective nodules in a wide range of *Lotus* hosts. To investigate the basis of this phenotype, we sequenced the complete genome of *Rl* Norway and compared it to the genome of the closely related strain *R. leguminosarum* bv. *viciae* 3841. The genome comprises 7,788,085 bp, distributed on a circular chromosome containing 63% of the genomic information and five large circular plasmids. The functionally classified bacterial gene set is distributed evenly among all replicons. All symbiotic genes (*nod*, *fix*, *nif*) are located on the pRLN3 plasmid. Whole genome comparisons revealed differences in the metabolic repertoire and in protein secretion systems, but not in classical symbiotic genes.

**Electronic supplementary material:**

The online version of this article (10.1186/s40793-018-0336-9) contains supplementary material, which is available to authorized users.

## Introduction

Legume crops are central to sustainable agricultural practices and food security [1, 2]. They have a low need for synthetic nitrogen fertilizers input, as they engage in a symbiosis with a group of diazotrophic bacteria collectively known as rhizobia. This symbiotic interaction is initiated by a molecular crosstalk between rhizobia and their cognate legume host. Upon recognition of specific signals, legume plants intracellularly accommodate rhizobia inside root organs called nodules, where they engage in a bidirectional nutrient exchange [3]. Occasionally, suboptimal interactions establish between the symbiotic partners. These lead to the formation of ineffective nodules in which limited to no nitrogen fixation occurs. These ineffective symbiotic associations are characterized by the formation of small white nodules, which results in reduced or no plant growth promotion [4].

Ineffective nitrogen-fixing symbioses have been described after introduction of crop legumes into areas where previously native legumes grew. The soil microbiota associated to native species can often outcompete inoculant strains [5]. For instance, ineffective nitrogen fixation occurs in fields where perennial and annual clovers co-exist [6, 7]. In field trials, inoculant strains were unable to completely overcome indigenous *R. leguminosarum* bv. *trifolii* strains and occupied on average 50% of the nodules [8]. In extreme cases, it has been shown that endogenous rhizobia can completely block the nodulation of introduced rhizobia. For example, the nodulation of pea cultivars Afghanistan and Iran by rhizobial inoculants is suppressed in natural soils by the presence of a non-nodulating strain [9]. However, although ineffective nodulation is a limiting factor for sustainable agriculture, the molecular basis underlying it remains largely unknown [10].

*Rhizobium leguminosarum* (*Rl*) strains are cognate micro-symbionts of legumes, including *Pisum*, *Lens*, *Lathyrus*, *Vicia*, *Phaseolus* and *Trifolium* [11]. However, a *R. leguminosarum* strain isolated from a *Lotus corniculatus* nodule in Norway exhibits a different compatibility range that includes several *Lotus* species and ecotypes. *Rl* Norway does not induce effective nodules in any *Lotus* species tested so far [12]. Strikingly, there are host genotype specific differences in the nodulation phenotypes induced by *Rl* Norway, as it cannot induce nodules on *L. japonicus* Gifu, but induces bumps on *L. japonicus* Nepal, and white nodules on *L. burttii* and *L. japonicus* MG-20. This is in contrast to compatible *Mesorhizobium* strains that induce monomorphic phenotypes in the same plant ecotypes [12].

The striking diversity of ineffective nodulation phenotypes induced by *Rl* Norway in *Lotus* motivated us to sequence and annotate its complete genome, and to compare it to the published genome of *R. leguminosarum* bv. *viciae* 3841 (*Rlv* 3841), a well-characterised *R. leguminosarum* strain. Here, we show that the genomes are largely conserved. There are no major differences in the *nif* and *fix* clusters required for nitrogen fixation and in the *nod* cluster essential for the production of nodulation factor. However, differences were observed in terms of metabolic and protein secretion system genes.

## Organism information

### Classification and features

**Fig. 1:**
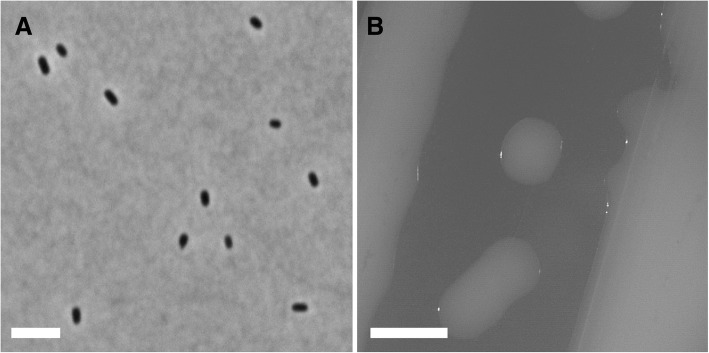
Morphological characterisation of *Rl* Norway. **a** Phase contrast micrograph of *Rl* Norway grown in liquid TY medium. Scale bar: 1 μm. **b** Photomicrograph of the colony morphology of *Rl* Norway grown on TY medium. Scale bar: 1 mm

*Rl* Norway is a Gram-negative strain in the order *Rhizobiales* of the class *Alphaproteobacteria* (Table 1). Cells are rod-shaped and have dimensions of 0.84 ± 0.11 μm in width and 1.43 ± 0.31 μm in length (Fig. 1a). This strain is fast growing and forms colonies after 3 days in TY medium at 28 °C. Colonies on TY are circular and slightly domed, their surface is shiny and smooth, and their texture is moderately mucoid (Fig. 1b).

**Table 1:** Classification and general features of *Rl* Norway in accordance to the MIGS recommendations [46] published by the Genome Standards Consortium [47]

MIGS ID	Property	Term	Evidence code^a^
	Classification	Domain Bacteria	TAS [48]
		Phylum *Proteobacteria*	TAS [49]
		Class *Alphaproteobacteria*	TAS [50, 51]
		Order *Rhizobiales*	TAS [50, 52]
		Family *Rhizobiaceae*	TAS [53–55]
		Genus *Rhizobium*	TAS [55–57]
		Species *Rhizobium leguminosarum*	TAS [55, 57–59]
	Gram stain	Negative	IDA
	Cell shape	Rod	IDA
	Motility	Motile	IDA
	Sporulation	Non-sporulating	NAS
	Temperature range	Mesophile	NAS
	Optimum temperature	28 °C	NAS
	pH range; Optimum	Not reported	
	Carbon source	Carbon sources sustaining growth are indicated in Figure S1	IDA
MIGS-6	Habitat	Soil, root nodule of *Lotus corniculatus*	TAS [12]
MIGS-6.3	Salinity	Not reported	
MIGS-22	Oxygen requirement	Aerobic	NAS
MIGS-15	Biotic relationship	Free-living/symbiont	TAS [12]
MIGS-14	Pathogenicity	Non-pathogen	NAS
MIGS-4	Geographic location	Norway	TAS [12]
MIGS-5	Sample collection	17. August 2006	TAS [12]
MIGS-4.1	Latitude	61°10′54.6″	TAS [12]
MIGS-4.2	Longitude	08°57′54.5″	TAS [12]
MIGS-4.4	Altitude	Not available	

^a^Evidence codes - *IDA* Inferred from Direct Assay, *TAS* Traceable Author Statement (i.e., a direct report exists in the literature), *NAS* Non-traceable Author Statement (i.e., not directly observed for the living, isolated sample, but based on a generally accepted property for the species, or anecdotal evidence). These evidence codes are from the Gene Ontology project [60]

**Fig. 2:**
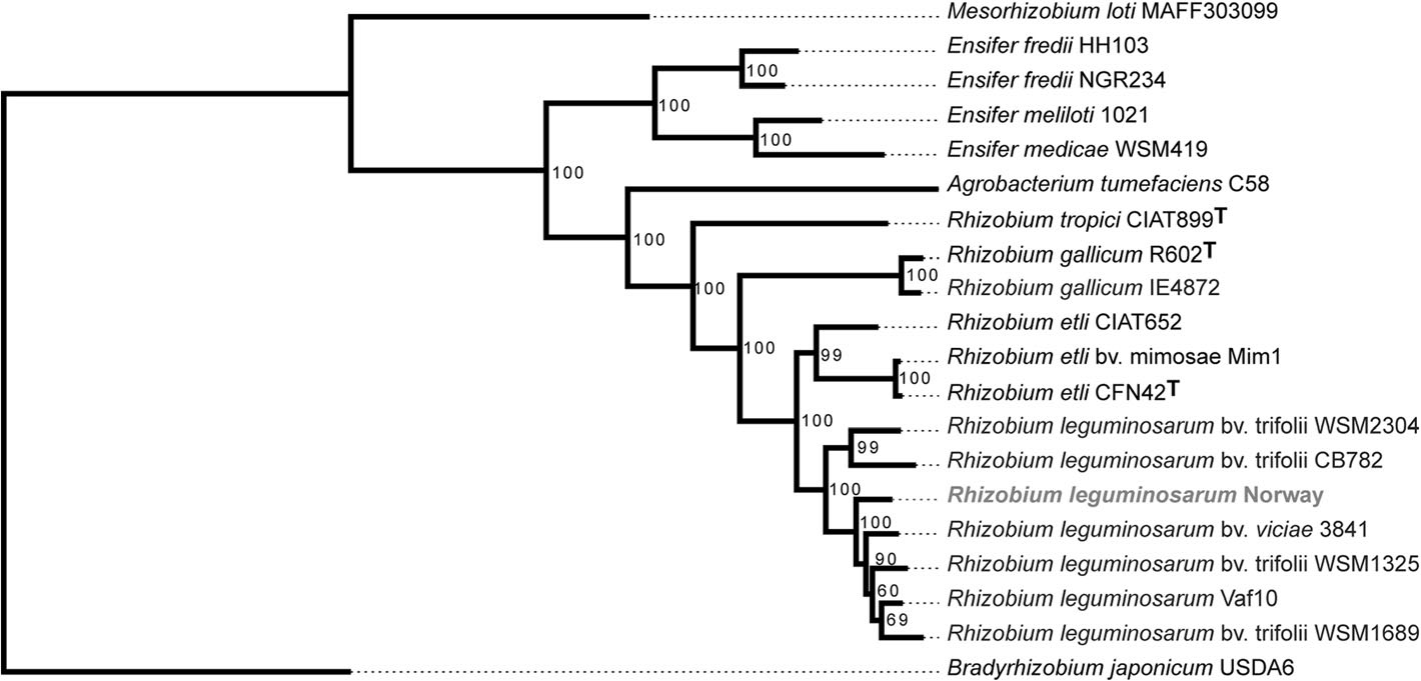
Phylogenetic tree showing the relationship between *Rl* Norway and other Rhizobia. The tree was constructed by maximum likelihood using the concatenated sequences of *recA*, *dnaK*, and *rpoB*. The calculated bootstrap values are indicated at the nodes. *Rl* Norway is highlighted in bold grey. Type strains are indicated with superscript ^**T**^. *B. japonicum* USDA6 was used as an out-group

The phylogenetic relationship of *Rl* Norway was inferred based on a concatenated tree of the *dnaK*, *recA,* and *rpoB* house-keeping genes (Fig. 2). Based on this phylogeny *Rl* Norway is placed within the *R. leguminosarum* group. The 16S rRNA gene of *Rl* Norway shows more than 99.9% identity with its orthologs in other *R. leguminosarum* strains, such as *Rlv* 3841 and *Rl* biovar *trifolii* WSM1325, WSM2304, and CB782.

The metabolic fingerprinting of *Rl* Norway was conducted with the Biolog GN2 MicroPlate. *Rl* Norway grew in multiple organic compounds as sole carbon source, these included Adonitol, L-Arabinose, D-Arabitol, D-Cellobiose, D-Fructose, and Glycerol, among others (Additional file 1: Figure S1). The metabolic fingerprinting of this strain was similar to the pattern described for other *R. leguminosarum* strains, but it was clearly distinct from the pattern of *Rlv* 3841 (Additional file 1: Figure S1) [13].

#### Symbiotaxonomy

*Rl* Norway was originally co-isolated from a *L. corniculatus* nodule together with two *Mesorhizobium* strains, but does not induce nodules in *L. corniculatus* or *L. japonicus* Gifu, when inoculated alone [12]. However, it induces bumps on *L. japonicus* Nepal, and ineffective nodules on *L. burttii* and *L. japonicus* MG-20 [12]. This polymorphic nodulation phenotype is not observed, when these hosts are inoculated with *Mesorhizobium* strains [12]. *Rl* Norway induces ineffective nodules in *Pisum, and Latyrus*. The nodulation and symbiotic characteristics of *Rl* Norway are summarized in Additional file 2: Table S1.

## Genome sequencing information

### Genome project history

*Rl* Norway was selected for sequencing, because of the striking diversity of ineffective nodulation phenotypes that it induces in *Lotus*, a host that belongs to a different cross-inoculation group. The complete genome sequencing was performed at the Genomics Service Unit (LMU Biocenter, Munich). The nucleotide sequences reported in this study have been deposited in the GenBank database under accession numbers CP025012.1, CP025013.1, CP025014.1, CP025015.1, CP025016.1, and CP025017.1. The data is summarized in Table 2.

**Table 2:** Genome sequencing project information for *Rl* Norway

MIGS ID	Property	Term
MIGS 31	Finishing quality	Finished
MIGS-28	Libraries used	Paired-end (Illumina); 1D Genomic (Nanopore)
MIGS 29	Sequencing platforms	Illumina MiSeq; Nanopore MinION
MIGS 31.2	Fold coverage	380×
MIGS 30	Assemblers	Unicycler v0.4.0
MIGS 32	Gene calling method	MicroScope
	Locus Tag	CUJ84
	Genbank ID	CP025012.1, CP025013.1, CP025014.1, CP025015.1, CP025016.1, and CP025017.1
	GenBank Date of Release	31. January 2018
	BIOPROJECT	PRJNA417364
MIGS 13	Project relevance	Agriculture, root nodule symbiosis
	Source Material Identifier	*Rhizobium leguminosarum* Norway

### Growth conditions and genomic DNA preparation

*Rl* Norway was grown at 28 °C and 180 rpm for 2 days in TY medium. Genomic DNA was isolated from 30 ml of a bacterial suspension (OD_600_ = 1.0) using the CTAB method [14]. The DNA quality was determined by nanodrop and gel electrophoresis.

### Genome sequencing and assembly

The genome was sequenced using a combination of Illumina and MinION sequencing technologies. Library construction and sequencing were performed at the Genomics Service Unit (LMU Biocenter, Munich). For whole genome sequencing a short read DNA library was generated with the Nextera Kit (Illumina) according to manufacturer’s instructions. Sequencing (2 × 150 bp, v2 chemistry) was performed on a MiSeq sequencer (Illumina) yielding around 15 Mio paired reads and 2.3 Gb of primary sequence. A long read library was prepared with the 1D Genomic DNA Sequencing Kit (Oxford Nanopores) according to manufacturer’s instructions. MinION (Oxford Nanopores) sequencing resulted in around 180,000 sequences with a total of 670 Mb primary sequence (mean length 3.8 kb). Hybrid genome assembly with Unicycler v0.4.0 [15] using default settings resulted in six circular contigs. The average base coverage of the genome is 380x.

### Genome annotation

Genome annotation was performed with RAST 2.0 [16, 17] and MicroScope [18]. Clusters of orthologous groups (COGs) of proteins were predicted using the COGNiTOR software [19], signal peptides were detected using the SignalP 4.1 server [20], and Pfam domains were predicted using the Pfam batch sequence search from EMBL-EBI [21]. Transmembrane predictions and CRISPR repeats were determined using the TMHMM Server v. 2.0 [22] and CRISPRFinder [23], respectively. All genes discussed in the text were manually inspected.

## Genome properties

**Fig. 3:**
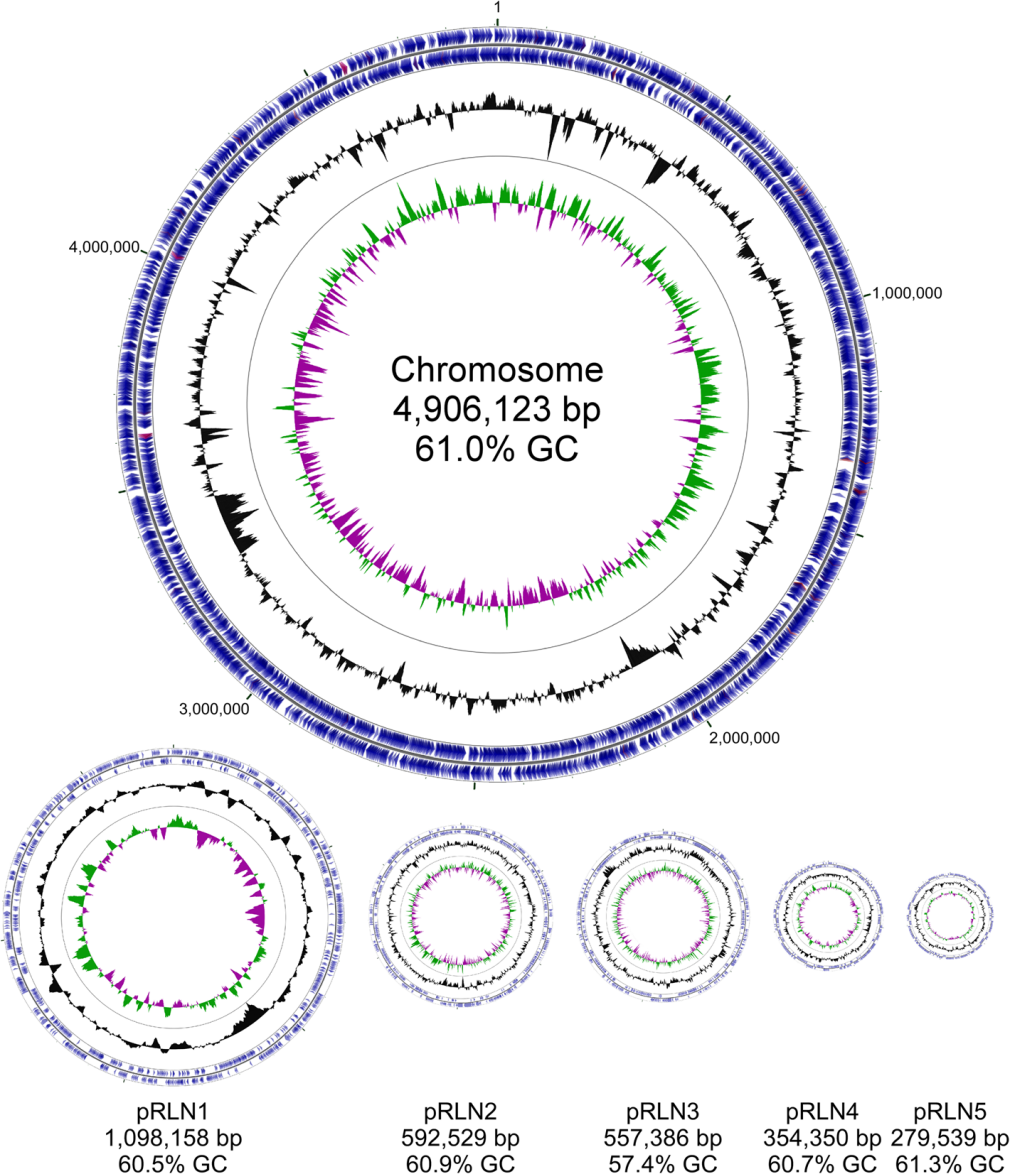
The chromosome and five plasmids of *Rl* Norway. The plasmids are depicted to scale with the chromosome one-half of this scale. The outermost circles show protein encoding genes (blue) and rRNA and tRNA genes (red) in clockwise and counter-clockwise orientation. The inner circles indicate deviations in GC content (black) and GC skew (green/purple). Plasmid maps were generated using GCView [61]

The genome of *Rl* Norway consists of 7,788,085 bp, distributed on a circular chromosome containing 63% of the genomic information and five large circular plasmids ranging from 280 to 1098 kb (Fig. 3). The complete genome and the chromosome are comparable in size to other *R. leguminosarum* strains [13, 24]. The chromosome contains three identical rRNA operons and 54 tRNA genes, none of which are found on any of the five plasmids (Table 3 and Fig. 3). In total 7866 protein-encoding genes were identified. BUSCO analysis [25] confirmed complete presence of the core bacteria dataset. The six replicons have a comparable mix of functional classes (Additional file 3: Figure S2A). However, all genes from the BUSCO core bacteria dataset are located on the chromosome, with only a few additional gene duplications on the plasmid replicons.

**Table 3:** Genome statistics for *Rl* Norway

Attribute	Value	%of Total
Genome size (bp)	7,788,085	100.00
DNA coding (bp)	6,859,686	88.08
DNA G + C (bp)	4,659,466	59.83
DNA scaffolds	6	100.00
Total genes	8079	100.00
Protein coding genes	7866	97.36
RNA genes	73	0.90
Pseudo genes	150	1.86
Genes in internal clusters	Not determined	Not determined
Genes with function prediction	6147	76.09
Genes assigned to COGs	6106	75.58
Genes with Pfam domains	6295	77.92
Genes with signal peptides	619	7.66
Genes with transmembrane helices	1656	20.50
CRISPR repeats	0	0.00

## Insights from the genome sequence

### Extended insights

The genomes of *Rl* Norway and *Rlv* 3841 have a very similar relative occurrence of functional protein encoding genes (Additional file 3: Figure S2B) and do not show any gross genomic alterations. Interestingly, although *Rl* Norway contains more protein encoding genes than *Rlv* 3841 (7866 vs. 7263 genes), the number of genes for which a functional annotation could be retrieved is almost identical (6106 vs. 6105 genes). Hence, the major difference lies in the number of not functionally classifiable genes (1760 vs. 1158 genes) (Table 4).

**Table 4:** Number of genes associated with general COG functional categories

Code	Value	%age	Description
J	210	2.67	Translation, ribosomal structure and biogenesis
A	0	0	RNA processing and modification
K	686	8.72	Transcription
L	219	2.78	Replication, recombination and repair
B	2	0.03	Chromatin structure and dynamics
D	40	0.51	Cell cycle control, Cell division, chromosome partitioning
V	74	0.94	Defense mechanisms
T	415	5.28	Signal transduction mechanisms
M	334	4.25	Cell wall/membrane biogenesis
N	92	1.17	Cell motility
U	106	1.35	Intracellular trafficking and secretion
O	199	2.53	Posttranslational modification, protein turnover, chaperones
C	342	4.35	Energy production and conversion
G	709	9.01	Carbohydrate transport and metabolism
E	831	10.56	Amino acid transport and metabolism
F	117	1.49	Nucleotide transport and metabolism
H	210	2.67	Coenzyme transport and metabolism
I	270	3.43	Lipid transport and metabolism
P	318	4.04	Inorganic ion transport and metabolism
Q	206	2.62	Secondary metabolites biosynthesis, transport and catabolism
R	905	11.51	General function prediction only
S	630	8.01	Function unknown
–	1760	22.37	Not in COGs

The total is based on the total number of protein coding genes in the genome

### Plasmid repertoire and genospecies classification

**Fig. 4:**
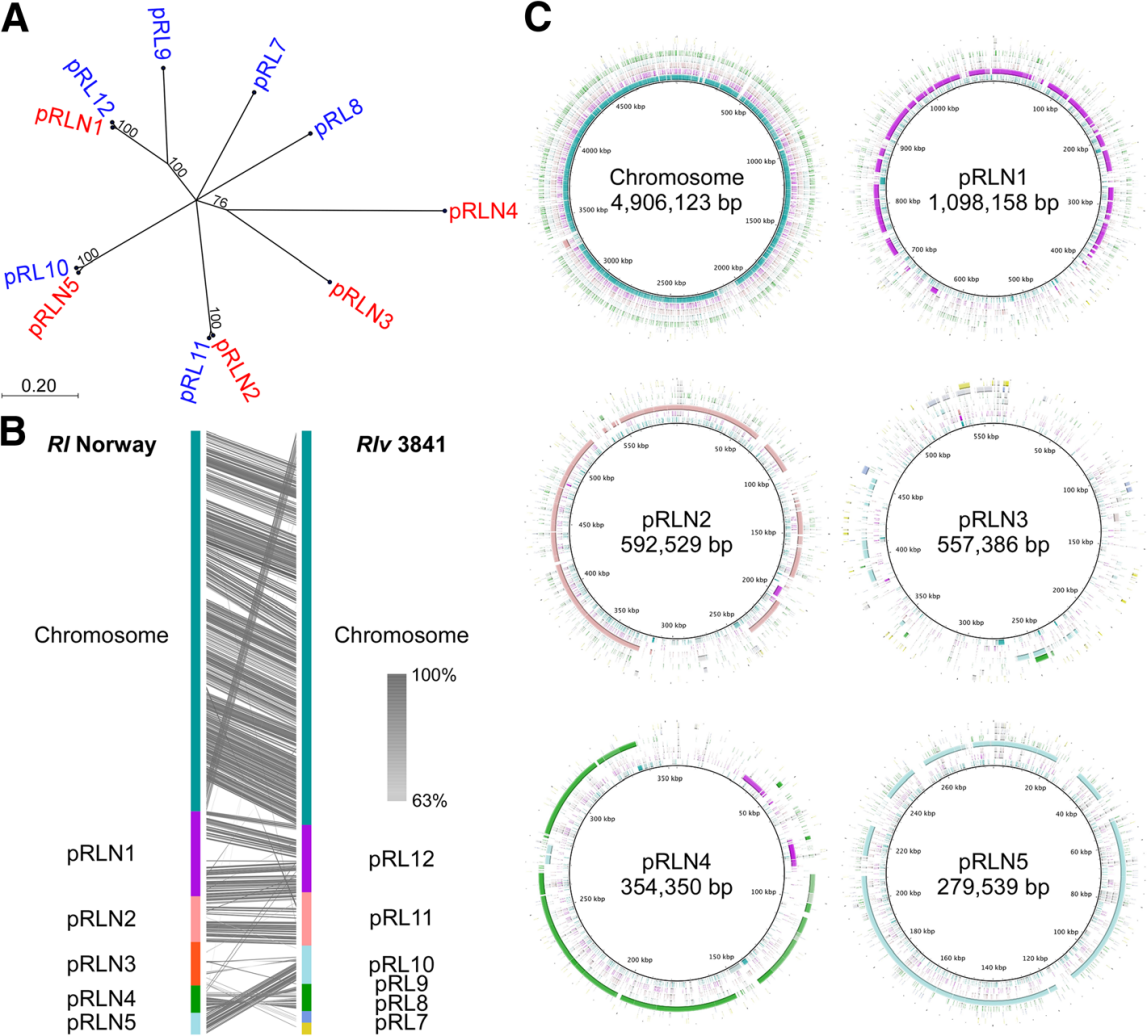
Genome comparison between *Rl* Norway and *Rlv* 3841. **a** Neighbor-joining tree of Rep proteins from both strains. Protein sequences for *RepA*, *RepB*, and *RepC* from the individual plasmids were aligned and the resulting alignments concatenated for analysis. *Rl* Norway proteins are depicted in red, *Rlv*3841 proteins in blue. Bootstrap values indicated on the nodes strongly support the relations between pRLN2 - pRL11, pRLN5 - pRL10, and pRLN1 - pRL12. Only bootstrap values > 70% are depicted. Branch lengths are given in terms of expected numbers of substitutions per nucleotide site. **b** For whole genome comparison the sequences of the chromosome and plasmids were concatenated for *Rl* Norway and *Rlv* 3841 and compared with BlastN in Easyfig 2.2.2 [62]. Levels of sequence identity are indicated by different shades of grey. **c** Gene contents comparison between the two strains. Depicted are the *Rl* Norway replicons and their respective homologous regions from the *Rlv* 3841 replicons. Plasmid maps were generated using BRIG [63]. Colors in the rings are the same as for the *Rlv* 3841 replicons in (**b**)

The five plasmids contain one set of putative *repABC* replication system genes each [26]. Comparative analysis of the Rep proteins from *Rl* Norway with those from *Rlv* 3841 revealed high identity between plasmids pRLN1 and pRL12, between pRLN2 and pRL11, and between pRLN5 and pRL10 (Fig. 4a). Gene content comparison and synteny analysis supported this result. Although large portions of pRLN4 and pRL9 are similar (Fig. 4b, and c), the RepABC proteins encoded in pRLN4 are more similar to their orthologs in pR132503.

Plasmid pRLN3 is slightly different than the other replicons of *Rl* Norway (Additional file 3: Figure S2A). It does not exhibit significant similarity to *Rlv* 3841 (Fig. 4b, and c), has a slightly lower GC content and a lower proportion of protein encoding sequences (Additional file 4: Table S2), and has a higher proportion of putative encoded proteins without known homologs (Additional file 3: Figure S2A). In addition, it is the only plasmid containing potentially active transposons (2 copies) and several incomplete and therefore most likely inactivated transposon copies. The pRLN3 RepABC proteins share high similarity to their orthologs in pRL1.

For genospecies classification, we compared the *Rl* Norway genome to representatives of the five proposed genospecies (gsA-gsE) [13]. Typically, genomes are regarded to belong to the same species if the ANI values are above 95%. The two highest average nucleotide identity (ANI) scores (*Rl* CC278f: 96.34%; *Rl* SM51: 95.59%) were found with members of the genospecies gsD. All other comparisons resulted in ANI scores below 95% (Table 5). The ANI score between *Rl* Norway and *Rlv* 3841, which belongs to gsB, is only 93.26%. Although genospecies gsA and *Rl* CC278f in gsD are not yet well supported [13], the results indicate that *Rl* Norway belongs to genospecies gsD. This also fits well with *Rl* Norway having a plasmid subtype combination typical for gsD strains ([13]& personal communication Peter Young).

**Table 5:** Genome comparison of *Rl* Norway with members of the five genospecies and the respective ANI scores

	Norway vs	One-way ANI 1	One-way ANI 2	Two-way ANI
(gsA)	WSM1325	93.45%	93.52%	93.70%
gsB	3841	93.01%	93.06%	93.26%
gsC	TA1	93.75%	93.80%	93.94%
gsD	SM51	95.40%	95.40%	95.59%
(gsD)	CC278f	96.11%	96.19%	96.34%
gsE	128C53	94.66%	94.75%	94.84%

### Central metabolism

In terms of central metabolic genes *Rl* Norway resembles *Rlv* 3841. Both strains harbour genes encoding enzymes of the tricarboxylic acid (TCA) cycle required for aerobic respiration and energy production [27], of the pentose phosphate pathway required for the oxidation of glucose and the synthesis of nucleotides [28], and of the Entner-Doudoroff pathway for the catabolism of glucose to pyruvate [29]. Both strains lack a gene encoding the phosphofructokinase, an essential enzyme of the Embden-Meyerhof-Parnas glycolysis. These genetic similarities were reflected in a similar growth pattern in different carbon sources using Biolog GN2 MicroPlates (Additional file 1: Figure S1) [13].

A noticeable difference in the Biolog assay was the assimilation of amino acids such as D- and L-alanine, L-serine and L-proline, and nucleosides. However, no major differences were observed in the genes mediating their metabolism. The only clear exceptions were that *Rl* Norway lacks a putative D-serine deaminase required for the conversion of D-serine to pyruvate, but contains two putative aspartate ammonia-lyases (CUJ84_pRLN3000095, CUJ84_pRLN3000303) and two putative asparagine synthetases (CUJ84_pRLN3000485, CUJ84_pRLN3000155). In terms of amino acid transport, two ABC-type broad specificity amino-acid transporters have been characterized in *Rlv* 3841, Aap (AapJQMP) and Bra (BraDEFGC) [30]. The *bra* (CUJ84_Chr003782–3787) and *aap* (CUJ84_Chr001810–1813) clusters are highly conserved in *Rl* Norway. Another interesting difference concerned the metabolism of butanoate. In contrast to *Rlv* 3841, *Rl* Norway did not grow on γ-hydroxybutyric acid (Additional file 1: Figure S1). This is supported by the lack of a gene cluster (pRL100133–138 in *Rlv* 3841) associated to γ-hydroxybutyrate utilisation [13]. Furthermore, *Rl* Norway harbours an ortholog to the *phbC1* gene (CUJ84_Chr001779), but lacks *phbC2*. These genes encode type I and type III poly-β-hydroxybutyrate (PHB) synthases, which are required for free-living and bacteroid PHB biosynthesis, respectively [31].

### Secretion systems

Gram-negative bacteria secrete a suite of proteins via macromolecular complexes that have been classified as type 1–6 secretion systems in addition to the *sec* and *tat* transport systems [32]. A survey of the *Rl* Norway genome indicates that this strain contains a large repertoire of secretion systems that is distinct from the repertoire of *Rlv* 3841 (Table 6). *Rl* Norway harbours five putative type 1 secretion systems (T1SS; Table 6). T1SSa, T1SSb and T1SSc are unique to *Rl* Norway. Interestingly, the genes encoding the T1SSa and T1SSc systems form operons with two large genes encoding putative repeats-in-toxin (RTX) toxins. The proteins forming the T1SSd and T1SSe have orthologs with more than 90% identity in *Rlv* 3841. For instance, the T1SSd proteins are orthologous to the PrsD and PrsE proteins of *Rlv* 3841 that are required for biofilm formation [33]. Like *Rlv* 3841, *Rl* Norway lacks T2SS and T3SS, but harbours T4SS and T6SS [34].

**Table 6:** Secretion system repertoire in *Rl* Norway

Secretion system	Location	Mandatory genes (gene identifier)
Type I secretion system (T1SS)		
T1SSa	Chromosome	*hlyD* (CUJ84_Chr000199), *hlyB* (CUJ84_Chr000200)
T1SSb	Chromosome	*hlyD* (CUJ84_Chr000279), *hlyB* (CUJ84_Chr000280)
T1SSc	Chromosome	*hlyD* (CUJ84_Chr002330), *hlyB* (CUJ84_Chr002331)
T1SSd	Chromosome	*prsE* (CUJ84_Chr003677), *prsD* (CUJ84_Chr003678)
T1Sse	Chromosome	*hlyD* (CUJ84_Chr004833), *hlyB* (CUJ84_Chr004834)
T4SSa	pRLN1	*virB1* (CUJ84_pRLN1000390), *virB2* (CUJ84_pRLN1000391), *virB3* (CUJ84_pRLN1000392), *virB4* (CUJ84_pRLN1000393), *virB5* (CUJ84_pRLN1000394), *virB6* (CUJ84_pRLN1000396), *virB8* (CUJ84_pRLN1000398), *virB9* (CUJ84_pRLN1000399), *virB10* (CUJ84_pRLN1000400)
Type 5 secretion system (T5SS)		
T5SSa	Chromosome	*autB* (CUJ84_Chr000739)
T5SSb	Chromosome	Partial *autB* (CUJ84_Chr002323)
T5SSc	pRLN2	*tpsA* (CUJ84_pRLN2000298), *tpsB* (CUJ84_pRLN2000297)
Type 6 secretion system (T6SS)		
T6SS	pRLN1	*tssB* (CUJ84_pRLN1000762), *tssC* (CUJ84_pRLN1000760, CUJ84_pRLN1000761), *tssD* (CUJ84_pRLN1000765), *tssE* (CUJ84_pRLN1000758), *tssF* (CUJ84_pRLN1000757), *tssG* (CUJ84_pRLN1000756), *tssH* (CUJ84_pRLN1000764), *tssI* (CUJ84_pRLN1000767), *tssK* (CUJ84_pRLN1000754), *tssL* (CUJ84_pRLN1000753), *tssM* (CUJ84_pRLN1000752)

Bacteria utilize T3SS, T4SS and/or T6SS to inject effector proteins directly into eukaryotic host cells or into other bacteria [35–37]. In rhizobia, these effectors can mediate compatibility with the host [38]. *Rl* Norway harbours a putative T4SS that is distinct from the T4SS from *Rlv* 3841. The respective T4SS encoding *virB* operons are not syntenic and the encoding genes share on average less than 30% identity. The T4SS of *Rl* Norway is encoded in the pRLN1 plasmid and is predicted to translocate proteins and not DNA, as *Rl* Norway lacks a VirD2 relaxase [39]. In addition, it has the peculiarity that the *virB11* gene is partially duplicated and two genes are located in-between the duplication.

*Rl* Norway and *Rlv* 3841 harbour syntenic *imp* (*tss*) and *hcp* clusters encoding type (i) T6SS. In both cases the *imp* cluster is lacking orthologs to the *evpJ* and *tssJ* genes. However, a comparison to *Agrobacterium tumefaciens* C58 revealed that these genes are also absent in the corresponding *imp* and *hcp* operons (atu4330-atu4352). In addition, all essential genes for protein secretion are conserved [40].

T5SS are structures in which the cargo protein translocates itself across the plasma membrane. These are classified into auto-transporters (translocator and cargo encoded in the same gene) and two-partner systems (translocator and cargo are encoded by two separate genes) [41]. *Rl* Norway harbours two T5SS auto-transporters. However, T5SSb is split into two genes and it is probably not a bona fide T5SS. *Rl* Norway also has one two-partner system, in which the cargo protein is a putative filamentous hemagglutinin (Table 6). In contrast, *Rlv* 3841 contains three auto-transporters, but no two-partner system [34].

### Symbiotic gene repertoire

Plasmid pRLN3 harbours all symbiotic genes in *Rl* Norway. The *nod* genes that are required for the synthesis and export of the nodulation factor, a key determinant in compatibility, are organised in one cluster (CUJ84_pRLN3000416–426) comprising the *nodJICBADFELMN* genes. They have the same organisation as the *nod* cluster in *Rlv* 3841 [24], and the encoded proteins share at least 93.6% identity with their *Rlv* 3841 orthologs. However, in contrast to *Rlv* 3841, *Rl* Norway lacks *nodO* and *nodT* orthologs in the proximity of the nod cluster. Interestingly, genes encoding putative transposases flank the *Rl* Norway *nod* cluster. The genes required for nitrogen fixation are located in proximity. The *fixABCX* (CUJ84_pRLN3000397–400) and the *nifAB* genes (CUJ84_pRLN3000401–402) are located almost directly downstream *nodJ*, whereas *nifNEKDH* (CUJ84_pRLN3000271–275), *fixSIHG* (CUJ84_pRLN3000258–261) and fix*PQON* (CUJ84_pRLN3000263–266) are located approximately 137.5 kb downstream of *nodJ*. The three subunits of the nitrogenase encoded by the *nifHDK* genes share 99.7, 93.5, and 96.3% identity to their respective *Rlv* 3841 orthologs. A noteworthy difference between both strains is that *Rl* Norway harbours a single *fixNOQP* operon encoding the essential cbb_3_ terminal oxidase, whereas *Rlv* 3841 contains two copies [24]. Furthermore, *Rl* Norway lacks genes encoding the FixK and FixL transcriptional regulators, which together with FnrN control the expression of the nitrogen fixation genes in other rhizobia strains [42]. Instead, *Rl* Norway harbours two putative *fnrN* genes (CUJ84_Chr002641, CUJ84_pRLN3000544) that are located in the chromosome and in the pRLN3 symbiotic plasmid. This is reminiscent of *R. leguminosarum* bv. *viciae* UPM791, in which FnrN is the global regulator of the fix genes. In this strain, FnrN is regulated by micro-aerobic conditions and binds a palindromic element called anaerobox [43, 44]. Putative anaerobox sequences were found upstream of *fnrN1* (CUJ84_Chr002641) and the *fixNOQP* and *fixGHIS* operons, which suggest that FnrN might regulate their expression in *Rl* Norway. However, no anaerobox was found upstream of *fnrN2* (CUJ84_pRLN3000544). Interestingly, *fnrN2* is approximately 16.5 kb upstream of a putative uptake hydrogenase cluster comprising 18 genes (CUJ84_pRLN3000511–528). The cluster organisation resembles the *hup* and *hyp* genes from *Rlv* UPM791 [45]. Notably, *Rlv* 3841 lacks such a hydrogenase cluster.

## Conclusions

Although detrimental in agriculture, ineffective nitrogen-fixing symbiosis remains poorly investigated. In this regard, *Rl* Norway is an interesting strain as it exhibits a parasitic behaviour in a wide range of hosts. Comparative genomic analyses with other *R. leguminosarum* strains have the potential to reveal novel factors mediating symbiotic compatibility and efficiency.

## Additional files

Additional file 1: **Figure S1.** *RI* Norway substrate utilization pattern determined by Biolog. In blue and yellow are indicated substrates only utilized by *RI* Norway and *Rlv* 3841, respectively. Green indicates substrates used by both strains, whereas white depicts conditions in which both strains did not grow. *Rlv* 3841 utilization pattern was extracted from [1]. (TIF 9702 kb)

Additional file 2: **Table S1.** Nodulation phenotypes of *Rl* Norway on selected hosts. (DOCX 68 kb)

Additional file 3: **Figure S2.** Distribution of functional classes of protein encoding genes within the *RI* Norway genome. (A) Functional class distribution across the six *RI* Norway replicons. (B) Comparison of the relative occurrence of functionally classified protein encoding genes between the *RI* Norway and *Rlv* 3841 genomes. Functional annotation (COG) was performed on WebMGA server [1]. (TIF 10046 kb)

Additional file 4: **Table S2.** Genome statistics for *Rl* Norway. (DOCX 47 kb)

## Abbreviations

COGs: Clusters of orthologous groups
CTAB: Cetyl trimethylammonium bromide
DAMPs: Damage associated molecular patterns
PHB: Poly-β-hydroxybutyrate
*Rl*: *Rhizobium leguminosarum*
RTX: Repeats-in-toxin
T1SS: Type 1 secretion system
TCA: Tricarboxylic acid
