# Supplementary figures and images for: Complete genome of Rhizobium leguminosarum Norway, an ineffective Lotus micro-symbiont
Source: Stand Genomic Sci. 2018 Dec 5;13:36. doi: 10.1186/s40793-018-0336-9 (PMC6280393; doi:10.1186/s40793-018-0336-9)

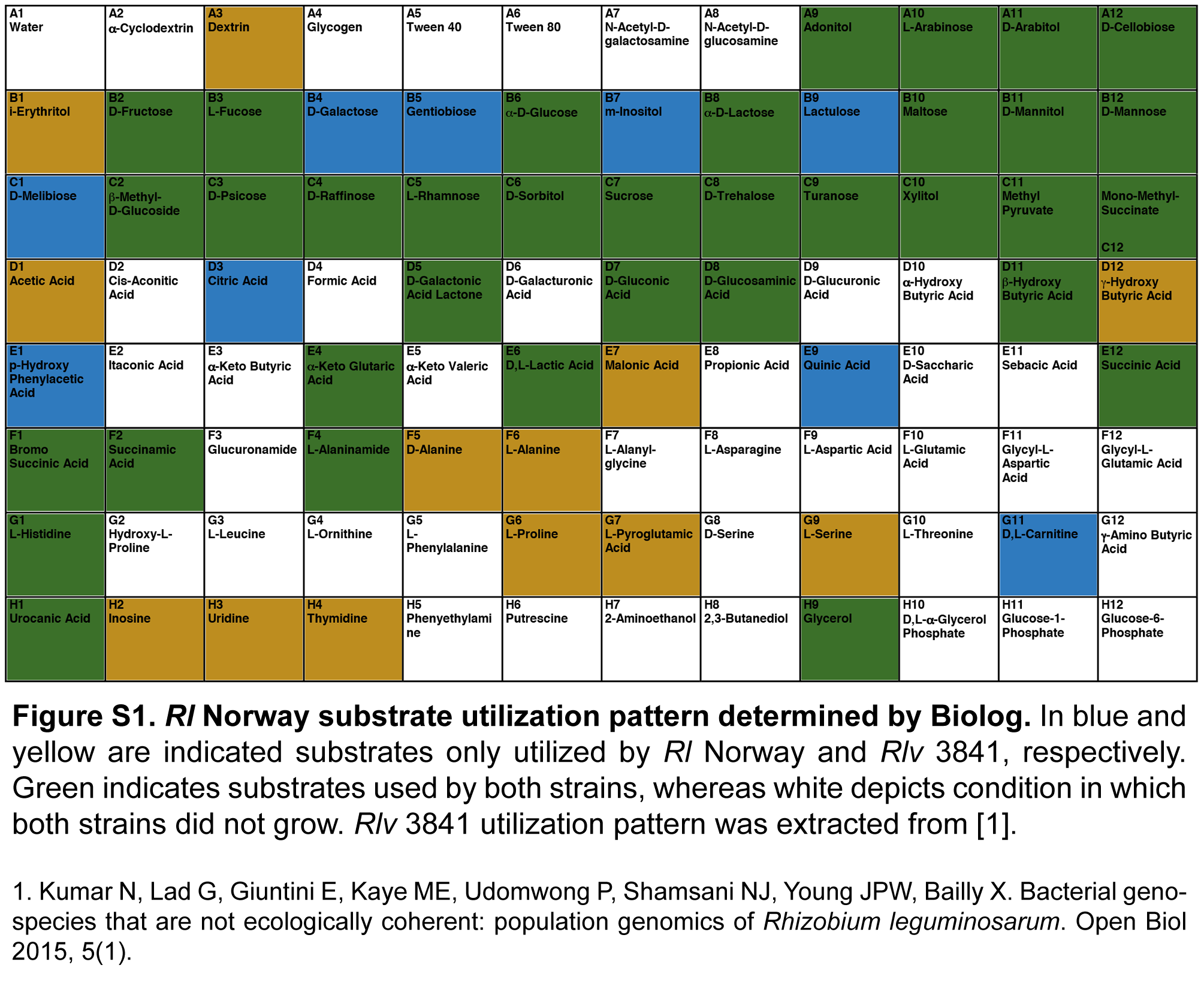

Supplement: Supplementary file 1 — Figure S1. RI Norway substrate utilization pattern determined by Biolog. In blue and yellow are indicated substrates only utilized by RI Norway and Rlv 3841, respectively. Green indicates substrates used by both strains, whereas white depicts conditions in which both strains did not grow. Rlv 3841 utilization pattern was extracted from [1]. (TIF 9702 kb) [file 40793_2018_336_MOESM1_ESM.tif]

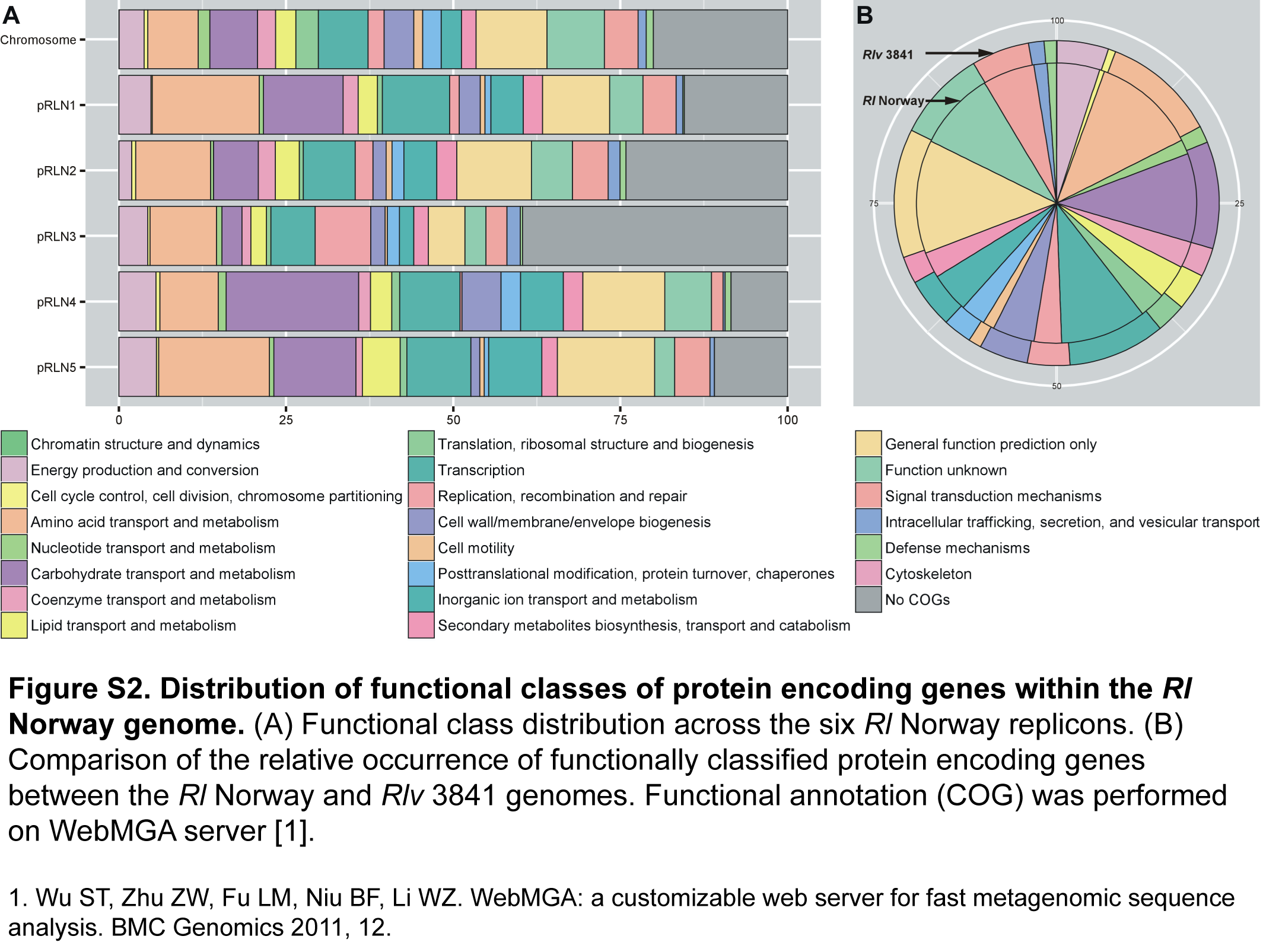

Supplement: Supplementary file 3 — Figure S2. Distribution of functional classes of protein encoding genes within the RI Norway genome. (A) Functional class distribution across the six RI Norway replicons. (B) Comparison of the relative occurrence of functionally classified protein encoding genes between the RI Norway and Rlv 3841 genomes. Functional annotation (COG) was performed on WebMGA server [1]. (TIF 10046 kb) [file 40793_2018_336_MOESM3_ESM.tif]
