# Supplementary material for: Complete genome of Rhizobium leguminosarum Norway, an ineffective Lotus micro-symbiont
Source: Stand Genomic Sci. 2018 Dec 5;13:36. doi: 10.1186/s40793-018-0336-9 (PMC6280393; doi:10.1186/s40793-018-0336-9)
Supplement: Supplementary file 2 — Table S1. Nodulation phenotypes of Rl Norway on selected hosts. (DOCX 68 kb) [file 40793_2018_336_MOESM2_ESM.docx]

**Table S1.** Nodulation phenotypes of *Rl* Norway on selected hosts.

| Species Name | Phenotype | Reference |
| --- | --- | --- |
| *L. japonicus* Gifu | Nod- | [1] |
| *L. japonicus* MG20 | Bump/Nod+Fix- | [1] |
| *L. japonicus* Nepal | Bump+Fix- | [1] |
| *L. filicaulis* | Nod- | [1] |
| *L. burttii* | Bump/Nod+Fix- | [1] |
| *L. pedunculatus* | Bump+Fix- | [1] |
| *L. glaber* | Tumor+/Fix- | [1] |
| *P. sativum* Sparkle | Nod- | [1] |
| *P. sativum Little Marvel* | Nod+Fix- | This work |
| *Latyrus sativus* | Nod+Fix- | This work |

1. Gossmann JA, Markmann K, Brachmann A, Rose LE, Parniske M. Polymorphic infection and organogenesis patterns induced by a *Rhizobium leguminosarum* isolate from *Lotus* root nodules are determined by the host genotype. *New Phytol* 2012, **196**(2):561-573.
