# Supplementary material for: Complete genome of Rhizobium leguminosarum Norway, an ineffective Lotus micro-symbiont
Source: Stand Genomic Sci. 2018 Dec 5;13:36. doi: 10.1186/s40793-018-0336-9 (PMC6280393; doi:10.1186/s40793-018-0336-9)
Supplement: Supplementary file 4 — Table S2. Genome statistics for Rl Norway. (DOCX 47 kb) [file 40793_2018_336_MOESM4_ESM.docx]

Table S2. Genome statistics for *Rl* Norway.

| Replicon | Size [base pairs] | GC content | Protein-encoding genes | Proportion coding sequences | Mean protein length  [amino acids] | rRNA operons | tRNA genes |
| --- | --- | --- | --- | --- | --- | --- | --- |
| Chromosome | 4,906,123 | 61.0% | 5045 | 87.6% | 284 | 3 | 54 |
| pRLN1 | 1,098,158 | 60.5% | 1079 | 90.8% | 308 |  |  |
| pRLN2 | 592,529 | 60.9% | 595 | 88.9% | 295 |  |  |
| pRLN3 | 557,386 | 57.4% | 570 | 83.5% | 272 |  |  |
| pRLN4 | 354,350 | 60.7% | 312 | 89.0% | 337 |  |  |
| pRLN5 | 279,539 | 61.3% | 265 | 90.2% | 317 |  |  |
| Total | 7,788,085 | 60.3% | 7866 | 88.3% | 302 | 3 | 54 |
